# Regression of choroidal metastasis from breast carcinoma with palbociclib

**DOI:** 10.1186/s40942-022-00398-w

**Published:** 2022-08-12

**Authors:** Shweta Parakh, Shrutanjoy Das, Shrey Maheshwari, Vinita Gupta, Gaurav Luthra, Saurabh Luthra

**Affiliations:** 1 Drishti Eye Institute, 16, Subhash Road, Astley Hall, Dehradun, 248001 Uttarakhand India; 2 AIIMS Rishikesh, Rishikesh, Uttarakhand India

**Keywords:** Choroidal metastasis, Breast carcinoma, Palbociclib, Cyclin-dependent kinase 4/6 (CDK4/6) inhibitor, Palliative chemotherapy

## Abstract

**Background:**

Uveal metastasis is reported to be the most common intraocular malignancy. The most common site of origin of ocular metastases in females is the breast. In some cases, uveal metastatic lesions respond to systemic chemotherapy. We report a case of a patient who developed choroidal metastasis, while on endocrine therapy with selective estrogen receptor modulator (SERM), tamoxifen, for estrogen receptor (ER) positive, progesterone receptor (PR) positive and (human epidermal growth factor receptor 2) HER2 negative primary breast carcinoma, which then regressed following systemic chemotherapy with palbociclib.

**Case description:**

An 83-year-old female, with a history of modified radical mastectomy, chemotherapy and radiation therapy for infiltrating duct carcinoma of the breast, presented with a choroidal metastatic lesion in the left eye along with liver and lung metastases, 3 years after the primary carcinoma was treated. At the time of presentation, she was on tamoxifen. The choroidal tumor showed regression after the introduction of palbociclib, a cyclin-dependent kinase 4/6 (CDK4/6) inhibitor.

**Conclusion:**

This report highlights the use of palbociclib, in the palliative treatment of choroidal metastasis from primary breast cancer. The use of chemotherapy for choroidal metastasis can help avoid external beam radiation therapy and its concurrent side effects. Although there are a few reports involving the use of palbociclib for metastatic breast carcinoma, all of those have been in conjunction with and/or following non-response to other treatment modalities. Ours is the first report wherein palbociclib has been used as the first-line palliative chemotherapy and helped in regression of choroidal metastasis.

## Background

Breast cancer is the most common neoplastic disorder diagnosed in females [1]. The most common site of origin of ocular metastases in females is the breast [2]. Uveal metastasis is reported to be the most common intraocular malignancy and often the first sign of tumor dissemination [3]. Uveal metastases frequently involve the choroid (88%) and uncommonly the iris (9%) or ciliary body (2%) [4]. The rich vascularity of the choroid and microenvironmental factors account for its highest metastatic efficiency index relative to all body tissues [5, 6].

Following surgical resection of the primary breast cancer, patients often receive adjuvant systemic chemotherapy with the aim of eradicating clinically and radiologically occult micrometastatic disease that may advance into frank metastatic disease if left untreated. Selection of adjuvant systemic therapies is based on classifying disease burden (number of lymph nodes, size of the primary tumor) and disease biology as determined by hormone receptor (HR) status [ER (estrogen receptor), PR (progesterone receptor)] and HER2 (human epidermal growth factor receptor 2) status, in addition to genomic assays [7]. Herein we report a case of an 83-year-old female who developed choroidal metastasis, while on endocrine therapy with selective estrogen receptor modulator (SERM), tamoxifen, for ER positive, PR positive and HER2 negative primary breast carcinoma, which then regressed following first-line palliative systemic chemotherapy with palbociclib.

## Case description

An 83-year-old female presented in August 2020 with complaints of gradual diminution of vision in the left eye (OS) for two weeks. She had a history of infiltrating duct carcinoma of the breast (Stage IIIA T2N2M0), for which she had undergone modified radical mastectomy followed by chemotherapy with epirubicin and radiation therapy in 2017. On immunohistochemistry, the carcinoma was found to be ER positive, PR positive and HER2 negative. At the time of presentation to us, she was on hormonal therapy in the form of oral tamoxifen 20 mg daily.

**Fig. 1:**
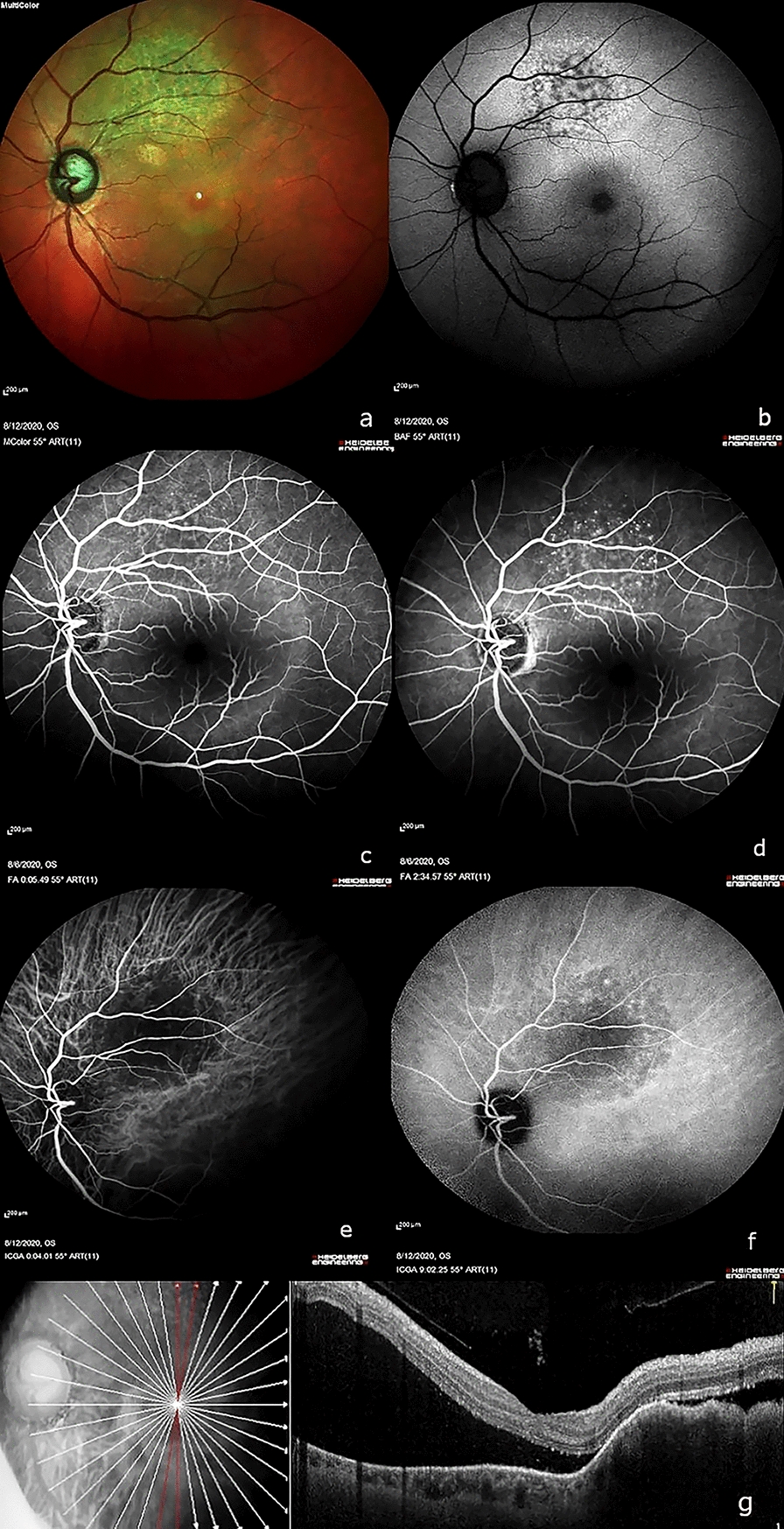
Multimodal imaging of choroidal metastasis in the left eye from primary breast carcinoma. At the first visit—**a** Multicolor fundus imaging showing a solitary yellowish elevated choroidal lesion along the superotemporal arcade with mottled hyperpigmentation on the surface. **b** Short wave fundus autofluorescence showing alternating areas of hyperautofluorescence and hypoautofluorescence within the lesion. **c**, **d** Fundus fluorescein angiography (FFA) showing early hypofluorescence with late hyperfluorescence and pinpoint leakage at the borders of the lesion. **e**, **f** Indocyanine green angiography (ICGA) showing early and late hypocyanescence. **g** Spectral domain optical coherence tomography (SD-OCT) showing an elevated choroidal hyporeflective lesion, with "lumpy-bumpy" overlying retinal pigment epithelium (RPE) and subretinal fluid with elongated "shaggy" photoreceptors

Best corrected visual acuity (BCVA) was 6/9 in the right eye (OD) and 6/24p in the left eye (OS). Intraocular pressure was normal in both eyes (OU). Anterior segment examination revealed pseudophakia OU. Fundus examination was within normal limits OD. The left eye showed a solitary yellowish elevated choroidal lesion along the superotemporal arcade, measuring approximately 3DD (disc diameters) by 3DD, with mottled hyperpigmentation on its surface (Fig. 1a). Subretinal fluid (SRF) was present at the macula. Autofluorescence (Heidelberg Retina Angiograph, Heidelberg Engineering, Heidelberg, Germany) showed alternate areas of speckled hyperautofluorescence and hypoautofluorescence within the lesion (Fig. 1b). Fundus fluorescein angiography (FFA) (Heidelberg Retina Angiograph, Heidelberg Engineering, Heidelberg, Germany) showed early hypofluorescence with late hyperfluorescence and pinpoint leakage at the borders of the lesion (Fig. 1c, d), while indocyanine green angiography (ICGA) showed early and late hypocyanescence. (Fig. 1e, f). Spectral domain optical coherence tomography (SD-OCT) (Optovue RTVue XR Avanti, Optovue Inc., Fremont, CA) revealed an elevated choroidal hyporeflective lesion, with thickened, undulating ("lumpy-bumpy") overlying retinal pigment epithelium (RPE). SRF with overlying elongated "shaggy" receptors were also seen (Fig. 1g). Bearing in mind her significant past history of breast carcinoma and the characteristic features on multimodal imaging, a provisional diagnosis of choroidal metastasis OS was made.

An urgent oncology clinic review was advised. Computed tomography (CT) scan of the chest and abdomen revealed mediastinal lymphadenopathy, right pleural effusion and multiple heterogeneously enhancing lesions in the liver, highly suggestive of metastases. Palliative chemotherapy for metastatic breast cancer was initiated by the oncologist in the form of oral palbociclib (cyclin-dependent kinase 4/6 (CDK4/6) inhibitor) 125 mg administered orally in 4-week cycles (3 weeks of treatment followed by 1 week off), in combination with oral letrozole (non-steroidal aromatase inhibitor) 2.5 mg daily (continuous treatment).

**Fig. 2:**
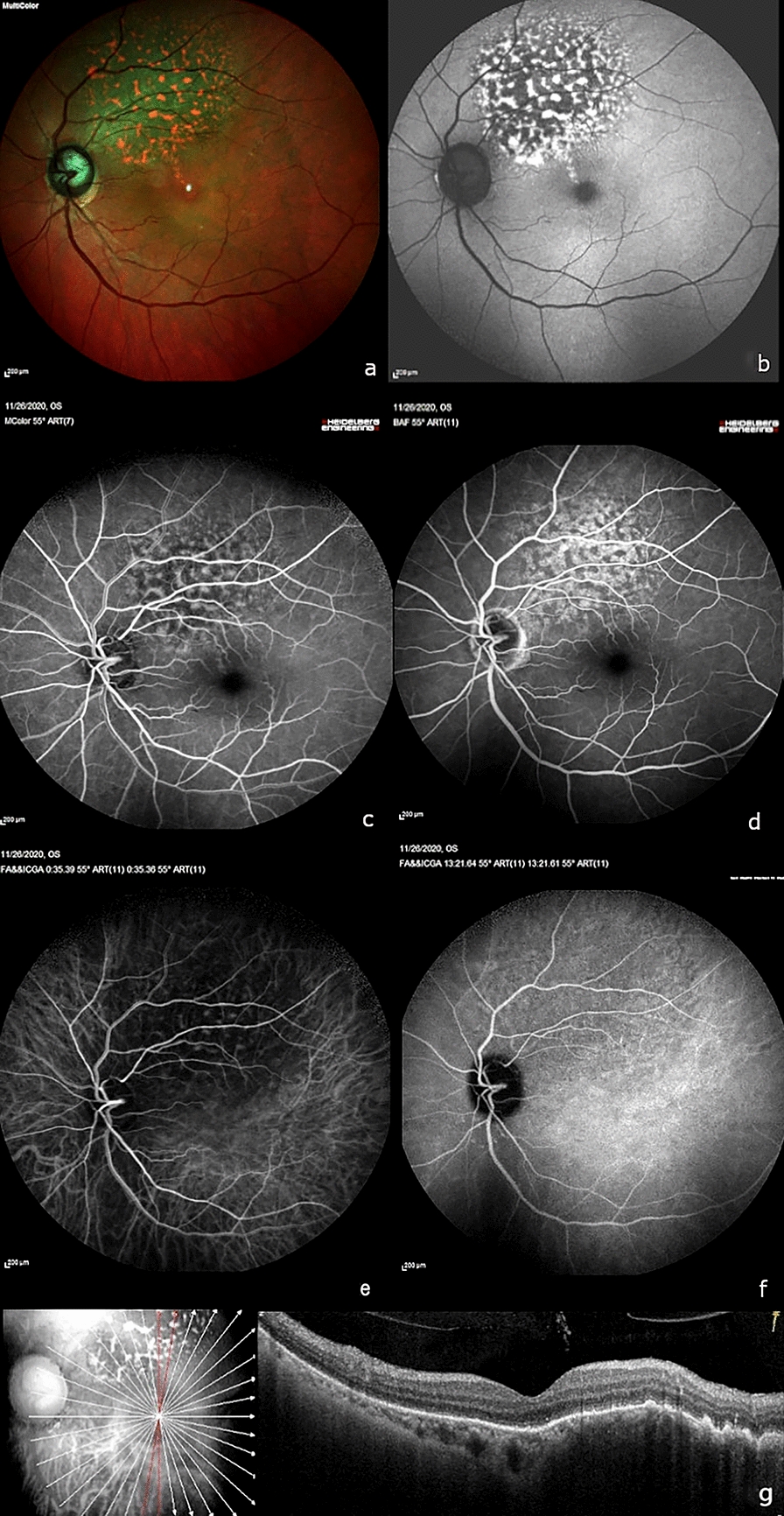
At the 3-month follow-up visit (post palliative chemotherapy with palbociclib)—**a** Multicolor fundus imaging showing flattening of the choroidal lesion and alteration in surface pigmentation. **b** Corresponding short-wave fundus autofluorescence showing prominent stippled hyperautofluorescence and hypoautofluorescence. **c**, **d** Fundus fluorescein angiography showing early hypofluorescence and late stippled hyperfluorescence. **e**, **f** Indocyanine green angiography (ICGA) showing normalization of hypocyanescence noted previously. **g** Spectral domain optical coherence tomography (SD-OCT) showing reduction in height of the choroidal hyporeflective lesion with resolution of overlying subretinal fluid

**Fig. 3:**
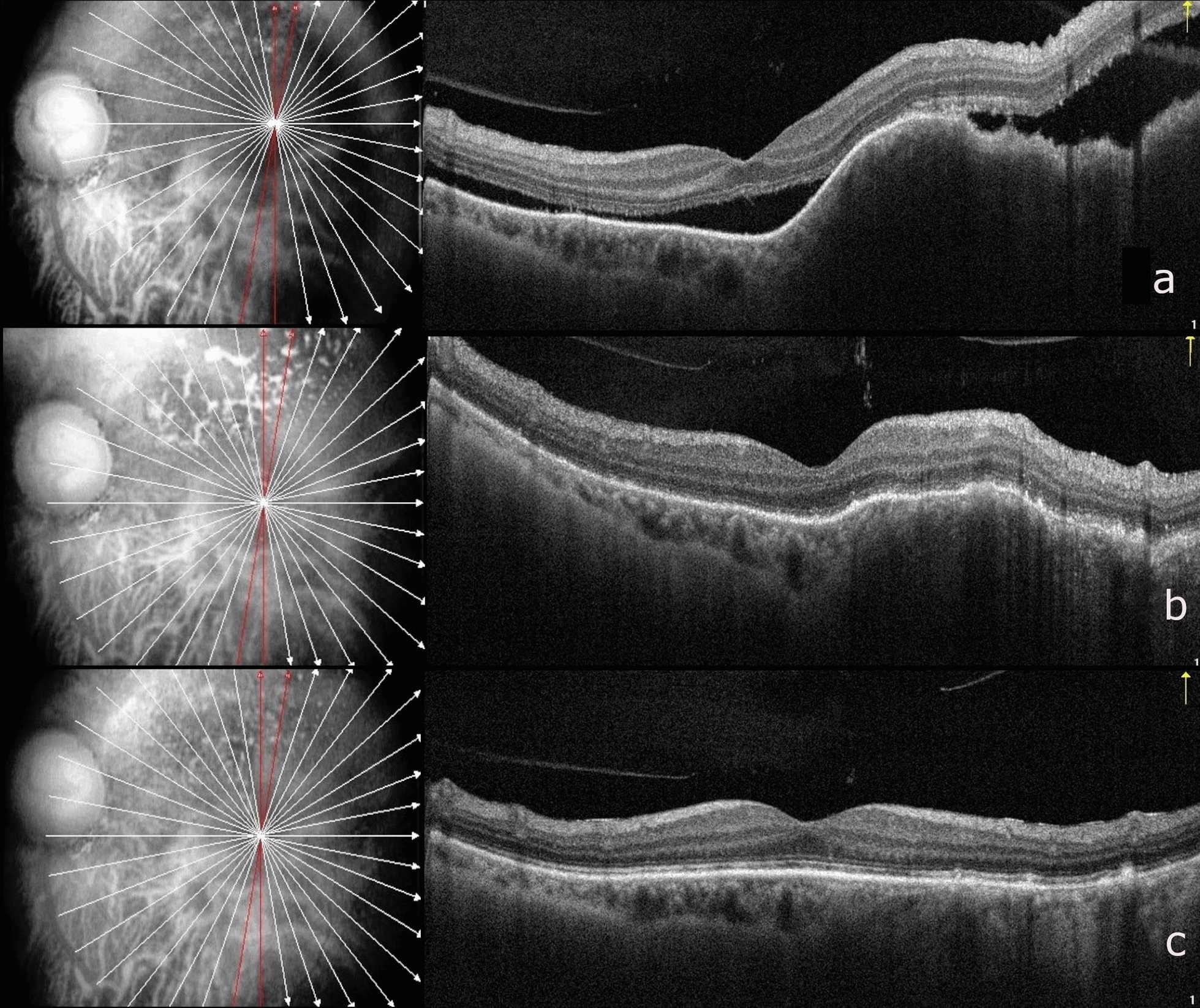
Serial optical coherence tomography (OCT) of our patient with choroidal metastasis in the left eye. **a** At the first visit, OCT showing an elevated choroidal hyporeflective lesion, with thickened, undulating, "lumpy-bumpy" overlying retinal pigment epithelium (RPE) and subretinal fluid (SRF) with elongated "shaggy" photoreceptors. **b** At the 3-week visit, after initiation of palbociclib, OCT showing reduction in height of choroidal lesion and regression of overlying SRF. **c** At the final visit (12-month follow-up), OCT showing flattening of the choroidal lesion and regressed SRF

Three weeks after initiation of the palliative chemotherapy, her BCVA improved to 6/9 OS. Although a clinically appreciable reduction in size of the choroidal lesion OS was not visible, the tumor surface showed RPE alteration. SD-OCT showed regression of subretinal fluid and reduction in height of the choroidal lesion. At the 3-month follow up visit, BCVA was maintained at 6/9 OS. Fundus examination revealed reduction in height of the choroidal lesion OS (Fig. 2a). Autofluorescence showed stippled hyperautofluorescence and hypoautofluorescence (Fig. 2b). Repeat FFA showed early hypofluorescence and late stippled hyperfluorescence (Fig. 2c, d). Repeat ICGA (Figs. 2e, f) showed regression of hypocyanescence that was noted prior to initiation of treatment. Corresponding resolution in SRF and reduction in height of the choroidal elevation was seen on OCT (Fig. 2g). Thus, a gradual regression of the choroidal lesion on OCT was documented over 12 months of follow-up (Fig. 3a–c). At the end of the year-long follow-up in the ophthalmology clinic, she maintained status quo.

## Discussion

The prognosis and treatment options of primary breast carcinoma are guided by its clinicopathological characteristics, the presence or absence of hormone receptors (HR), the expression of the HER2 oncogene and multiparameter genomic assays [7]. 75% of breast carcinomas are ER and PR positive, and such tumors demonstrate a good response to endocrine therapy [7, 8]. SERMs such as tamoxifen are used in premenopausal and postmenopausal women; aromatase inhibitors (anastrozole, letrozole, and exemestane) are only used in postmenopausal women and are generally preferred over tamoxifen as adjuvant therapy, but may also be prescribed successively with tamoxifen [9]. Our postmenopausal patient developed a choroidal metastasis in an ER positive, PR positive and HER2 negative infiltrating duct carcinoma while on hormone therapy with tamoxifen. The aim of treatment being largely palliative at this advanced stage, the patient was offered conservative management in the form of palbociclib.

Management of patients with choroidal metastases depends on their systemic status in addition to the number, location, multifocality and laterality of the ocular tumors. Treatment options comprise of systemic chemotherapy, immunotherapy, hormone therapy, whole eye radiotherapy, plaque radiotherapy, indocyanine green augmented transpupillary thermotherapy (TTT), anti-VEGF injections, photodynamic therapy (PDT), surgical resection and enucleation [10]. It has been reported in literature that uveal metastatic lesions may respond to systemic chemotherapy [11–15], with corresponding regression of subretinal fluid documented on OCT [16, 17]. This may reduce the need for local radiation therapy [13, 15].

On 31st March, 2017, the U.S. Food and Drug Administration (FDA) granted regular approval to palbociclib (IBRANCE®, Pfizer Inc.) for the treatment of ER positive, PR positive and HER2 negative advanced or metastatic breast cancer in combination with an aromatase inhibitor as initial endocrine-based therapy in postmenopausal women [18, 19]. The PALOMA-2 [A Study of Palbociclib (PD-0332991) + Letrozole vs. Letrozole For 1st Line Treatment Of Postmenopausal Women With ER + /HER2- Advanced Breast Cancer] and PALOMA-3 [Palbociclib (PD-0332991) Combined With Fulvestrant In Hormone Receptor + HER2-Negative Metastatic Breast Cancer After Endocrine Failure] clinical trials have proven that concomitant use of palbociclib to standard endocrine therapy significantly improved outcomes in treatment of ER-positive, HER2-negative advanced breast cancer [20, 21]. There have been a few reports involving the use of palbociclib in conjunction with other treatment modalities for metastatic breast carcinoma. In a case series of 3 patients with late-onset choroidal metastasis that presented more than 20 years after diagnosis of the primary breast cancer, one of the patients was administered palbociclib for slowly progressive hepatic metastases [22]. Arya and Duker reported the regression of a choroidal metastatic lesion after the initiation of vinorelbine following failure to respond to various treatment modalities. The primary tumor was Stage IIIA T3N1M0 infiltrating duct carcinoma of the breast with ER positive, PR positive, HER2 negative expressivity. The primary malignancy was treated with surgical resection and adjuvant chemoradiation, followed by hormone therapy with numerous agents (including palbociclib) in combination with kinase inhibitors for ER resistance [23]. In another case report of ER positive, PR positive, HER2 negative breast carcinoma in a 38-year-old woman treated with right mastectomy and axillary lymph node dissection, followed by four cycles of docetaxel and cyclophosphamide and adjuvant radiation, the patient developed multiple bone metastases in addition to mediastinal lymphadenopathy, two years after the primary diagnosis [24]. The patient underwent bilateral salpingo-oophorectomy and was started on gemcitabine with denosumab. Two months later, she developed bilateral choroidal metastases, which regressed following treatment with four intravitreal injections of bevacizumab 1.25 mg and orbital radiation of 37.5 Gy in 14 fractions. After completion of bevacizumab and radiation therapy, their patient was started on palbociclib and letrozole—unlike our patient who responded to palbociclib and letrozole as first-line chemotherapy for the choroidal metastasis. In a recent case series, Beddok et al., reported that concomitant administration of palbociclib with locoregional and/or symptomatic irradiation at a metastatic site was reasonably well tolerated in a cohort of 30 women with metastatic breast cancer [25].

## Conclusion

Infrequent spontaneous regression of choroidal metastatic lesions, without any therapy directed towards the primary tumor, has also been reported, although rare [26]. However, in our case, since regression of subretinal fluid and reduction in size of the choroidal lesion coincided with the initiation of palbociclib, it was the most probable contributor to the observed clinical response. The use of chemotherapy for choroidal metastasis can help avoid external beam radiation therapy and its concurrent side effects such as exposure keratopathy, radiation retinopathy, and optic neuropathy [27].

Previous reports involving the use of palbociclib for metastatic breast carcinoma have been in conjunction with and/or following non-response to other treatment modalities [22–25]. Ours is the first report wherein palbociclib has been used as the first-line palliative chemotherapy and helped in regression of choroidal metastasis. Therefore, we suggest the use of palbociclib as the initial palliative chemotherapy for choroidal metastasis following breast carcinoma.

## Data Availability

The datasets used and/or analyzed during the current study are available from the corresponding author on reasonable request.

## Abbreviations

HR: Hormone receptor
ER: Estrogen receptor
PR: Progesterone receptor
HER2: Human epidermal growth factor receptor 2
SERM: Selective estrogen receptor modulator
BCVA: Best corrected visual acuity
OD: Right eye
OS: Left eye
OU: Both eyes
FFA: Fundus fluorescein angiography
ICGA: Indocyanine green angiography
SD-OCT: Spectral-domain optical coherence tomography
CT: Computed tomography
CDK: Cyclin-dependent kinase
TTT: Transpupillary thermotherapy
PDT: Photodynamic therapy
PALOMA-2: A Study of Palbociclib (PD-0332991) + Letrozole vs. Letrozole For 1st Line Treatment Of Postmenopausal Women With ER + /HER2- Advanced Breast Cancer
PALOMA-3: Palbociclib (PD-0332991) Combined With Fulvestrant In Hormone Receptor + HER2-Negative Metastatic Breast Cancer After Endocrine Failure
